# Loss of the Anorexic Response to Systemic 5-Aminoimidazole-4-Carboxamide-1-β-D-Ribofuranoside Administration Despite Reducing Hypothalamic AMP-Activated Protein Kinase Phosphorylation in Insulin-Deficient Rats

**DOI:** 10.1371/journal.pone.0071944

**Published:** 2013-08-14

**Authors:** Kaio F. Vitzel, George Bikopoulos, Steven Hung, Rui Curi, Rolando B. Ceddia

**Affiliations:** 1 School of Kinesiology and Health Science, York University, Toronto, ON, Canada; 2 Department of Physiology and Biophysics, Institute of Biomedical Sciences, University of Sao Paulo, SP, Brazil; Hosptial Infantil Universitario Niño Jesús, CIBEROBN, Spain

## Abstract

This study tested whether chronic systemic administration of 5-aminoimidazole-4-carboxamide-1-β-D-ribofuranoside (AICAR) could attenuate hyperphagia, reduce lean and fat mass losses, and improve whole-body energy homeostasis in insulin-deficient rats. Male Wistar rats were first rendered diabetic through streptozotocin (STZ) administration and then intraperitoneally injected with AICAR for 7 consecutive days. Food and water intake, ambulatory activity, and energy expenditure were assessed at the end of the AICAR-treatment period. Blood was collected for circulating leptin measurement and the hypothalami were extracted for the determination of suppressor of cytokine signaling 3 (SOCS3) content, as well as the content and phosphorylation of AMP-kinase (AMPK), acetyl-CoA carboxylase (ACC), and the signal transducer and activator of transcription 3 (STAT3). Rats were thoroughly dissected for adiposity and lean body mass (LBM) determinations. In non-diabetic rats, despite reducing adiposity, AICAR increased (∼1.7-fold) circulating leptin and reduced hypothalamic SOCS3 content and food intake by 67% and 25%, respectively. The anorexic effect of AICAR was lost in diabetic rats, even though hypothalamic AMPK and ACC phosphorylation markedly decreased in these animals. Importantly, hypothalamic SOCS3 and STAT3 levels remained elevated and reduced, respectively, after treatment of insulin-deficient rats with AICAR. Diabetic rats were lethargic and displayed marked losses of fat and LBM. AICAR treatment increased ambulatory activity and whole-body energy expenditure while also attenuating diabetes-induced fat and LBM losses. In conclusion, AICAR did not reverse hyperphagia, but it promoted anti-catabolic effects on skeletal muscle and fat, enhanced spontaneous physical activity, and improved the ability of rats to cope with the diabetes-induced dysfunctional alterations in glucose metabolism and whole-body energy homeostasis.

## Introduction

Insulin is a key player in the control of intermediary metabolism with widespread effects on food intake, body composition, and whole-body energy homeostasis [1]. Under conditions of insulin deficiency, such as in type 1 diabetes (T1D), a myriad of complex and interactive dysfunctional metabolic alterations take place [2]. Pronounced loss of muscle and fat mass, hyperphagia, polydipsia, polyuria, hyperglycemia, and ketoacidosis are typical manifestations of insulin deficiency found in T1D [1], [2]. The administration of exogenous insulin corrects these dysfunctional metabolic alterations and remains the main therapeutic approach for the treatment of T1D [1], [2]. However, the challenge in the therapy of T1D is to precisely match insulin administration with dietary carbohydrate intake to tightly maintain euglycemia in the long-term [2]. Therefore, alternative pharmacological approaches have been tested either alone or in combination with insulin in an attempt to improve glycemic control and prevent multiple complications of diabetes [3]. In this context, several studies have investigated the effectiveness of pharmacological agents that activate the cellular energy sensor AMP-activated protein kinase (AMPK) in diabetes therapy [4]–[6]. AMPK is a heterotrimeric enzyme that can be allosterically and covalently [7], [8] activated under conditions of metabolic stresses (e.g. exercise, glucose deprivation, etc) that increase intracellular AMP, ADP or Ca^2+^ [7].

In its activated state, AMPK turns on catabolic pathways that increase ATP synthesis while simultaneously suppressing energy-consuming biosynthetic pathways in an attempt to restore the energy charge of cells [7]. A drug extensively used to study the effects of acute and chronic AMPK activation in various cells and tissues *in vitro* or *in vivo* is the AMP analog AICAR, which induces AMPK activation without altering the intracellular AMP:ATP ratio [9]. In peripheral tissues such as skeletal muscle, AICAR-induced AMPK activation has been extensively demonstrated to increase glucose uptake, glycogen synthesis, and fatty acid oxidation [3]–[5], [10]–[14]. When administered directly into the brain, AICAR induces hypothalamic AMPK activation which, in turn, phosphorylates/de-activates its downstream target acetyl CoA carboxylase (ACC), leading to an orexigenic effect [15]–[17]. Interestingly, we have previously demonstrated that intraperitoneal (i.p.) administration of AICAR reduced hypothalamic AMPK phosphorylation and increased ambulatory activity and whole-body energy expenditure in lean rats. Additionally, a significant reduction in food intake was observed in these animals [18]. These findings provided evidence that antagonistic effects on food intake could be achieved depending on whether AICAR was centrally or systemically administered. Moreover, the anorexic response to exogenous leptin has been reported to be exacerbated in rats chronically receiving i.p. AICAR injections [18], providing evidence that systemically administered AICAR actually causes a leptin-sensitizing effect.

Leptin exerts anorexic effects through mechanisms that involve inhibition of AMPK activation in the hypothalamus [19]. Upon binding to its receptors, leptin induces phosphorylation of STAT3, which then undergoes homodimerization and translocation into the nucleus. Through this cascade of events, leptin enhances and suppresses the expression of anorexigenic and orexigenic genes, respectively [19]. It also promotes the expression of SOCS3, which serves as a negative feedback regulator of leptin signaling. Therefore, while activation of AMPK in the hypothalamus induces food intake and favors weight gain, inhibition of this kinase by leptin blocks these centrally-mediated effects on energy balance [19], [20]. Based on these observations, we hypothesized that systemically administered AICAR could actually attenuate the hyperphagia that is typically associated with insulin deficiency [2]. This could be achieved through AICAR-induced inhibition of AMPK activation and reduction of SOCS3 content in the hypothalamus, as previously reported by us [18]. These centrally-mediated effects of systemic AICAR administration could also alter body composition and whole-body energy homeostasis in a way that ameliorates dysfunctional alterations of T1D. In order to test this hypothesis, rats were rendered diabetic through the administration of the pancreatic-β-cell-toxic drug streptozotocin [21]. Diabetic animals with almost undetectable levels of circulating insulin and severely hyperglycemic were then treated for 7 consecutive days with AICAR as recently described by us [14]. The results of this study provide novel evidence that AICAR triggered selective anti-catabolic effects on skeletal muscle and adipose tissue with important implications for spontaneous physical activity and whole-body energy balance in diabetic rats. Furthermore, analysis of hypothalamic content and phosphorylation of proteins involved in leptin signaling revealed molecular mechanisms underlying the differences in food intake response found between control and diabetic rats upon systemic AICAR administration.

## Materials and Methods

### Animals

Male albino rats from the Wistar strain (Charles River Laboratories, Montreal, Quebec, Canada) weighing 180–200 g (initial weight) were used in all experiments. The animals were housed in cages with free access to water and standard rat chow (60, 27, and 13% of calories from carbohydrate, protein, and fat, respectively, energy density 3.43 kcal/g, TestDiet Richmond, IN). The animals were maintained in a constant-temperature (23°C), with a fixed 12-h light, 12-h dark cycle (07∶00–19∶00 h). The protocol containing all animal procedures described in this study were specifically approved by the Committee on the Ethics of Animal Experiments of York University (York University Animal Care Committee, YUACC, permit number: 2011-14) and performed strictly in accordance with the YUACC guidelines. All surgery was performed under Ketamine/Xylazine anesthesia, and all efforts were made to ensure that the animals did not suffer unduly during and after the experimental procedure.

### Reagents

AICAR was purchased from Toronto Research Chemicals (Toronto, Ontario). STZ was obtained from Sigma (St. Louis, MO). Specific antibodies against AMPK, P-AMPK (Thr172), ACC, STAT3, P-STAT3, SOCS3, and GAPDH were purchased from Cell Signaling Technology Inc. (Beverly, MA). The anti-P-ACC (Ser 79) antibody was purchased from Millipore (Temecula, CA).

### STZ-induced Diabetes

Male Wistar rats (180–200 g) were fasted overnight and then rendered diabetic by a single intraperitoneal (i.p.) injection of STZ (65 mg/kg BW). Following the STZ injection, animals had *ad libitum* access to food and water. Fourty eight hours post-STZ injection, blood was drawn from the saphenous vein to measure glycemia using the LifeScan OneTouch Ultra glucometer. Only animals eliciting plasma glucose levels equal or greater than 25 mmol/l were used in this study. This was confirmed by measuring glycemia twice a week in all animals as recently described by us [14].

### Animal Groups and AICAR Treatment

In order to investigate the effects of chronic pharmacological AMPK activation on body composition and whole-body energy balance, three weeks after STZ-induced diabetes, the animals were further divided into the following 4 groups: Control, AICAR, STZ, and STZ+A. Control (Con) animals were those non-diabetic animals originally injected with saline instead of STZ that received single daily saline injections for 7 consecutive days. The AICAR (A) group consisted of rats that were non-diabetic controls that received single daily IP AICAR injections (400 mg/kg BW) for 7 consecutive days. The STZ and STZ+A groups consisted of those rats originally rendered diabetic by STZ injections and while the former group received single daily IP injections of saline, the latter group received single daily IP injections of AICAR (same dose as the AICAR group) for 7 consecutive days. The AICAR dose selected was based on previous unpublished experiments in which we detected the minimum amount of the compound necessary to consistently induce AMPK activation in peripheral tissue (e.g. skeletal muscles, adipose tissue, and liver), as well as to cause an anorexic response that was accompanied by reductions in hypothalamic AMPK phosphorylation in rats [14], [18]. In fact, this study is a continuation of a recently published study in which we used the exact same AICAR dose and tested its effects on muscle metabolism and glycemic control in insulin-deficient rats [14]. AICAR or saline was always injected between 09∶30 and 10∶00 h. At the end of the AICAR-treatment period, the animals were anaesthetized for tissue extraction and then immediately euthanized [14].

### Determination of Body Weight, Food Intake, Water Intake, and Feed Efficiency

Body weight (BW) and food intake were monitored on a daily basis. The animals were weighed everyday between 09∶30 and 10∶00 h and then the chow pellets remaining in the cages were weighed. Food intake was also monitored once by using the comprehensive laboratory animal monitoring system (CLAMS; Columbus Instruments, Inc. Ohio, USA) and the values were identical to those obtained by manual determinations. The CLAMS is also equipped with an automated system that measures volumetric drinking, which allowed us to monitor water intake in individual cages. Feed efficiency was estimated by dividing the gain of BW (g) by the energy consumed (kcal) over a specific time-period [22]. In this study, feed efficiency was calculated during 7 days that preceded AICAR injection and also during the 7-day AICAR injection-period and expressed as g/kcal/7 days.

### Animal Dissection for Body Composition Analysis and Determination of LBM

At the end of the AICAR-treatment period, all rats were weighed, anesthetized (Ketamine/Xylazine 90 mg and 10 mg/100 g B.W., respectively), decapitated, and exsanguinated. The abdominal and thoracic cavities were then longitudinally incised and the internal organs were exposed. The liver, kidneys, heart, were carefully removed and individually weighed. Next, retroperitoneal (Retro) and subcutaneous (SC) inguinal (Ing) fat pads as well as all remaining visible fat were thoroughly removed and weighed. To assess the weight of the viscera, the stomach and intestines were isolated and weighed separately. A longitudinal anterior skin incision from neck to tail was then made. A scalpel was used to detach the entire skin consisting exclusively of fur and subcutaneous white adipose tissue (WAT) from the carcass of each animal. A similar procedure was carried out to remove the skin from the head. The head and body skins were weighed separately. The mass of the carcass consisting of skeletal muscle and bones combined with the skinned head, heart, kidneys, and liver was used as LBM [23].

### Determination of *in vivo* Metabolic Parameters

The CLAMS was also used to perform all automated *in vivo* determinations as previously described [23]. Briefly, the CLAMS measures oxygen consumption (VO_2_), carbon dioxide production (VCO_2_), and respiratory exchange ratio (RER). Each cage is also equipped with a system of infrared beams that detects animal movement in the X and Z axes, which was used to determine spontaneous ambulatory activity. Energy expenditure (heat) was calculated by multiplying the calorific value (CV = 3.815+1.232**×**RER) by VO_2_. Measurements using the CLAMS were performed during the last day of the AICAR-treatment period. The animals were placed in the CLAMS at 09∶00 immediately following saline or AICAR injections. The first hour of data collected in the CLAMS was discarded, since it is the time required for the rats to fully acclimatize to the cage environment [23]. The rats were monitored for a 24 h-period encompassing the light (07∶00–19∶00 h) and dark (19∶00–07∶00 h) cycles.

### Determination of Circulating Insulin and Leptin

Blood from all animals was collected by saphenous vein bleeding and the serum was used to determine insulin (ELISA kit, Cat # EZRMi-13k, from Millipore, Billerica, MA, USA) and leptin (ELISA Rat leptin kit, Cat # 90040 from Crystal Chem Inc. Downers Grove, IL, USA). All procedures were performed according to instructions provided by the manufacturers of the kits.

### Determination of Content and Phosphorylation of Proteins by Western Blot

Immediately after decapitation, the hypothalamus was dissected using the optic tracts, the thalamus, and the mammillary body as landmarks [18], and then quickly frozen in liquid nitrogen and stored at −80°C. The tissues were homogenized in a buffer containing 25 mM Tris-HCl and 25 mM NaCl (pH 7.4), 1 mM MgCl2, 2.7 mM KCl, 1% Triton-X, and protease and phosphatase inhibitors (0.5 mM Na_3_VO_4_, 1 mM NaF, 1 µM leupeptin, 1 µM pepstatin, and 20 mM PMSF). Homogenates were centrifuged, the infranatant collected, and an aliquot was used to measure protein concentration by the Bradford method. Samples were diluted 1∶1 (vol/vol) with 2 × Laemmli sample buffer, heated to 95°C for 5 min, and subjected to SDS-PAGE. All primary antibodies were used in a dilution of 1∶1,000 except for P-AMPK (1∶500). Equal loading was confirmed by both GAPDH detection and Ponceau staining of all membranes.

### Statistical Analysis

Data were pooled from two independent experiments (N = 8 animals per group). Results are presented as means ± SEM. One- and Two-way ANOVA followed by Tukey’s multi-comparison post-hoc tests were used to assess differences among groups. Differences were considered statistically significant at P<0.05.

## Results

### Food Intake, BW, Feed Efficiency, and Water Intake

**Figure 1:**
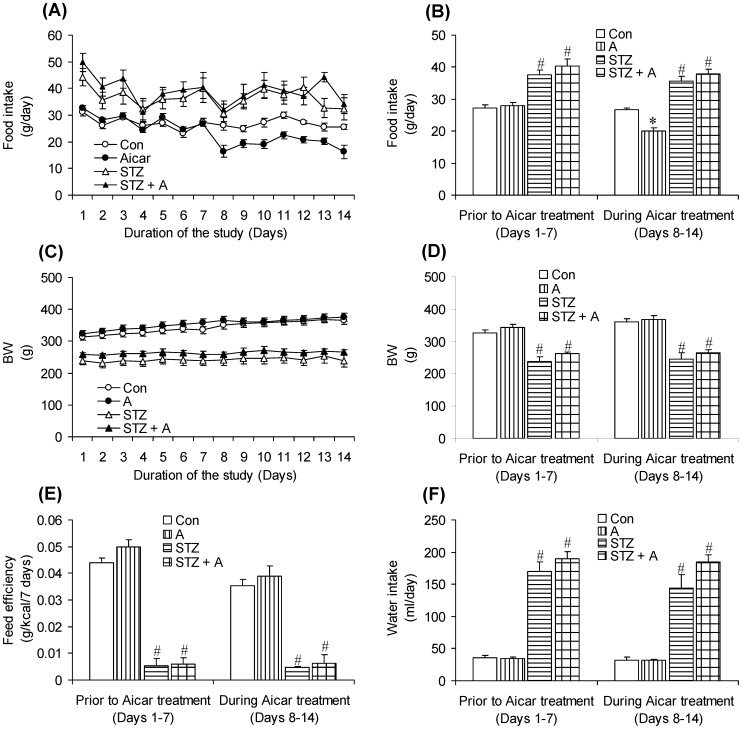
Polyphagia and polydipsia of Streptozotocin (STZ)-induced diabetes is not affected by AICAR treatment. Time-course of food intake (A) average food intake (B), time-course body weight (BW) (C), average BW (D), feed efficiency (E), and water (F) intake of control (Con, saline-injected), AICAR-injected (A), STZ, and STZ plus AICAR (STZ+A) rats. Time-course graphs show data from animals monitored on a daily basis one week prior to (days 1–7) and during AICAR administration (days 8–14). *P<0.05 vs. Con, STZ, and STZ+A. ^#^P<0.05 vs. Con and A (Two-way ANOVA, N = 8).

Food intake did not differ between control (27.18±0.94 g/day) and AICAR (27.87±1.10 g/day) rats during the week that preceded AICAR administration (Figure 1A and B). However, food intake was consistently reduced by ∼25% in rats injected with AICAR (20.17±0.85 g/day) when compared to controls (26.65±0.65 g/day) (Figure 1A and B). As expected, STZ rats were hyperphagic and elicited ∼1.4-fold increase in food intake when compared to controls. Hyperphagia was not altered in STZ rats treated with AICAR (Figure 1A and B). Body weight of control and AICAR rats progressively increased throughout the study from 314.08±9.37 g and 324.50±8.97 g to 364.92±11.31 and 375.61±11.50, respectively. At the week prior to AICAR administration, all diabetic rats (STZ and STZ+AICAR) had significantly lower (∼25%) body weights than the non-diabetic counterparts (control and AICAR rats), a condition that did not change during the AICAR administration-period (Figure 1C and D). In fact, STZ and STZ+AICAR rats did not gain any significant amount of weight throughout the study (Figure 1C and D) and feed efficiency was very low in diabetic rats either prior to or during AICAR administration (Figure 1E), demonstrating that the fuel ingested was not diverted towards growth or accretion of fat mass. Water intake did not differ between control and AICAR-treated rats either prior to (39.97±3.35 and 34.56±1.84 ml/day) or after AICAR administration (Figure 1F). However, this variable was increased by ∼4.7- to 5.5-fold in diabetic rats either prior to (170.32±14.44 and 189.37±11.19 ml/day) or after AICAR administration (144.30±20.40 and 184.96±11.19 ml/day) when compared to control animals (Figure 1F). Hyperphagia and polydipsia are hallmarks of T1D [2] and are in line with the severe hyperglycemia and insulin deficiency that these animals demonstrated [14].

### Body Composition

**Figure 2:**
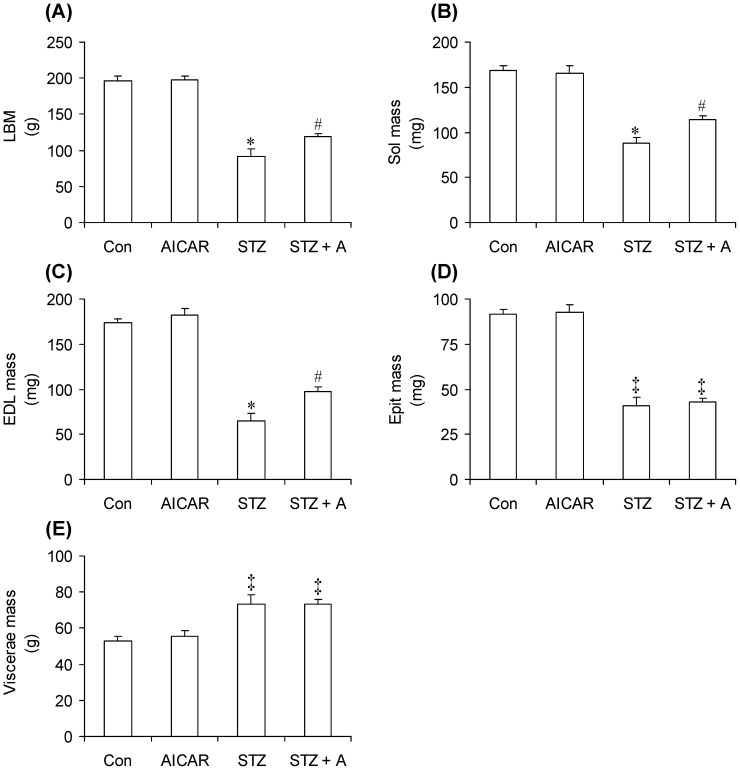
Treatment with AICAR attenuates the catabolic effects of streptozotocin (STZ)-induced diabetes on body composition. Lean body mass (LBM) (A), soleus (Sol) (B), extensor digitorum longus (EDL) (C), and epitrochlearis (Epit) (D) muscles, and viscera (E) masses were measured in control (Con, saline-injected), AICAR-injected (A), STZ, and STZ plus AICAR (STZ+A) rats at the end of the study. *P<0.05 vs. Con, A, and STZ+A. ^#^P<0.05 vs. Con, A, and STZ. ^‡^P<0.05 vs. Con and A. (One-way ANOVA, N = 8).

**Figure 3:**
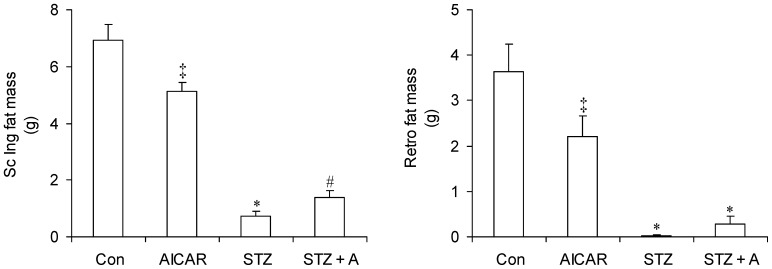
Fat depot-specific anti-catabolic effect of AICAR in rats with streptozotocin (STZ)-induced insulin deficiency. Subcutaneous (Sc) inguinal (Ing) fat (A) and retroperitoneal (Retro) fat (B) masses of control (Con, saline-injected), AICAR-injected (A), STZ, and STZ plus AICAR (STZ+A) rats were extracted and quantified at the end of the study. *P<0.05 vs. Con, A, and STZ+A. ^#^P<0.05 vs. Con, A, and STZ. ^‡^P<0.05 vs. Con, STZ, and STZ+A (One-way ANOVA, N = 8).

While LBM did not differ between control (196.42±5.96 g) and AICAR-injected (197.72±5.55 g) rats, this variable was significantly reduced by ∼54% in STZ rats. AICAR also had an LBM-sparing effect, since in STZ+A rats this variable was only ∼40% lower than controls and significantly higher (∼30%) than that of STZ rats (Figure 2A). In order to assess the contribution of oxidative and glycolytic skeletal muscles to the alterations in LBM seen in STZ and STZ+A rats, we measured the masses of soleus (Sol), extensor digitorum longus (EDL), and epitrochlearis (Epit) muscles in all animals. These muscles were chosen because of their wide range of reported fiber-type distributions. The percentages of type I, type IIa, and type IIb in Sol, EDL, and Epit muscles are 84/16/0, 3/57/40 [24], and 15/20/65 [25], respectively. The masses of the Sol (Figure 2B), EDL (Figure 2C), and Epit (Figure 2D) muscles did not differ between control and AICAR-treated rats. However, in STZ rats the masses of all three muscles were significantly reduced by 48% (Sol), 63% (EDL), and 56% (Epit) when compared to controls. Interestingly, AICAR treatment significantly attenuated the muscle-wasting effect of STZ-induce insulin deficiency in Sol (Figure 2B) and EDL (Figure 2C), but not in Epit (Figure 2D) muscles. In fact, the masses of the Sol and EDL muscles of STZ rats treated with AICAR were 30% and 46% lower than controls and significantly higher (∼30% and 50%) than that of STZ rats (Figures 2B and 2C), respectively. Because STZ rats were hyperphagic and AICAR reduced food intake in control rats (Figure 1A), we also measured the mass of the viscera in all animals. This variable was found to be 35–40% increased in STZ and STZ+A rats (Figure 2E). The significant increased viscera mass of STZ rats was essentially because of the accumulated undigested humidified chow in the gastrointestinal tract of these hyperphagic and polydipsic animals (Figure 2E). Assessment of adipose tissue mass also revealed that SC Ing and Retro fat pads were reduced by 36% and 40% in AICAR-treated rats, respectively, when compared to controls (Figure 3A and B). In STZ rats, the masses of SC Ing and Retro fat pads were drastically reduced, reaching values that corresponded to ∼10.5% (Figure 3A) and ∼0.5% (Figure 3B) of their controls, respectively. Interestingly, while in non-diabetic rats AICAR reduced fat mass, in STZ rats AICAR treatment attenuated fat loss; however, this occurred in a depot-specific manner. In fact, while AICAR-treated STZ rats had values of SC Ing fat mass ∼1.9-fold (1.39±0.25 g vs. 0.73±0.16 g) higher than those of STZ rats (Figure 3A), no significant differences were found with regards to Retro fat pad masses between STZ and STZ+A rats (0.02±0.01 g vs. 0.29±0.16 g) (Figure 3B). We have also recently reported that AICAR administration attenuated fat loss in the epidydimal fat depot of STZ rats [14].

### VO_2_, RER, Heat, and Ambulatory Activity

**Figure 4:**
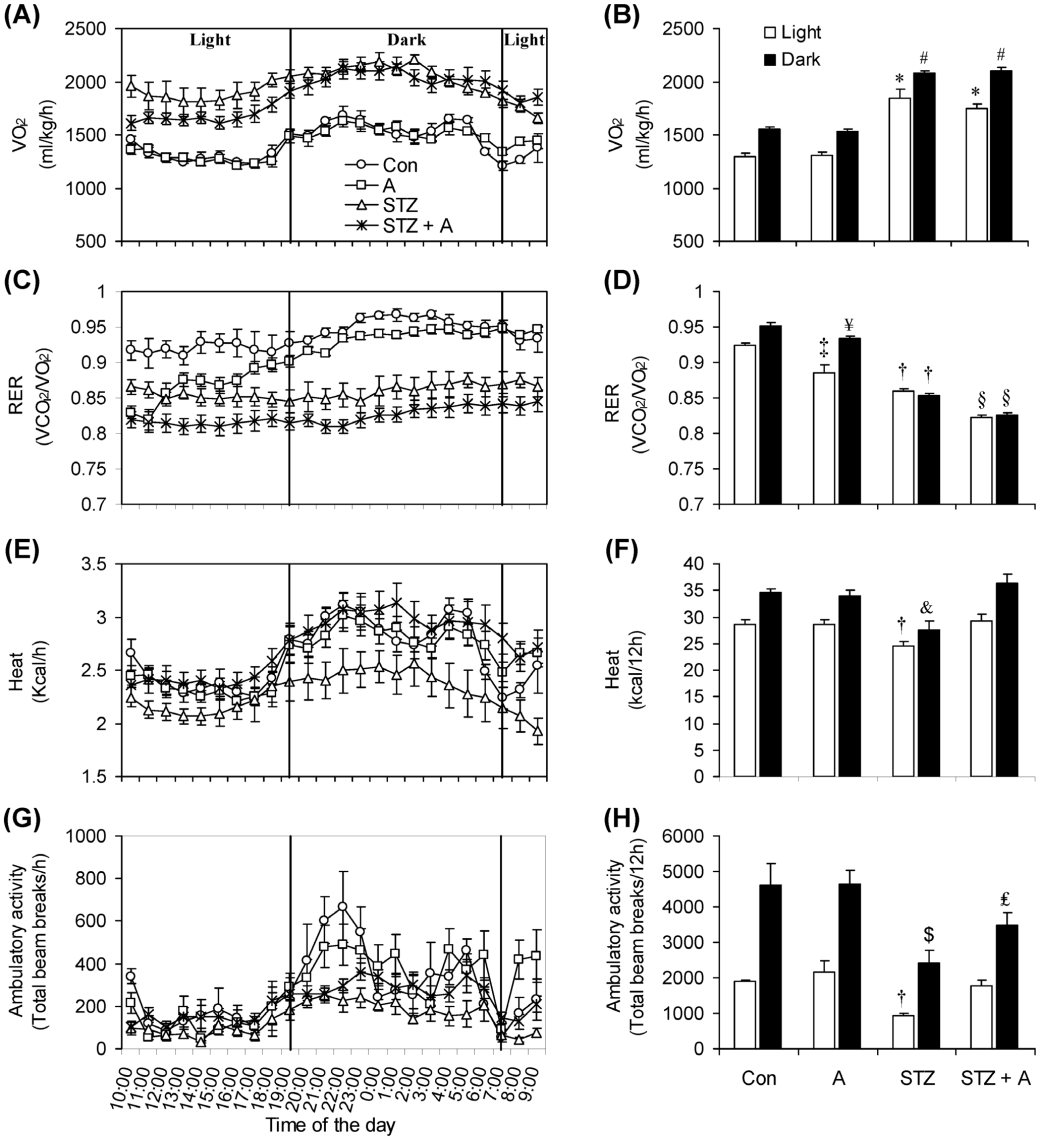
Profile of oxygen consumption (VO_2_) (A and B), respiratory exchange ratio (RER) (C and D), energy expenditure (heat) (E and F), and ambulatory activity (G and H) in control (Con, saline-injected), AICAR-injected (A), streptozotocin (STZ) diabetic, and streptozotocin plus AICAR (STZ+A) rats. At the end of the AICAR administration-period all animals were placed in the CLAMS for 24 h. *P<0.05 vs. Con and A light. ^#^P<0.05 vs. Con and A dark. ^‡^P<0.05 vs. Con light. ^¥^P<0.05 vs. Con dark. ^†^P<0.05 vs. Con and A light. ^§^P<0.05 vs. Con, A, and STZ light and dark. ^&^P<0.05 vs. Con, A, and STZ+A dark. ^$^P<0.05 vs. Con, A, and STZ+A dark. ^£^P<0.05 vs. Con, A, and STZ dark (Two-way ANOVA, N = 8).

Given the profound changes that occurred in food intake and body composition of STZ rats and the effects of AICAR treatment on these variables, we assessed major parameters involved in the regulation of whole-body energy metabolism. In this context, no significant differences were found for VO_2_ between control and AICAR-treated rats during the light and dark cycles (Figure 4A and B). However, in STZ rats this variable was significantly increased by 43% and 34% during the light and dark cycles, respectively, when compared to control rats. Similar increments in VO_2_ during the dark and light cycles were also found in STZ+A rats, indicating that the effects of STZ-induced insulin deficiency were not affected by AICAR treatment (Figure 4A and B). Analysis of RER revealed that, on average, AICAR reduced this variable from 0.924±0.004 to 0.885±0.01 during the light cycle and from 0.952±0.003 to 0.933±0.004 during the dark cycle. However, the RER-reducing effect of AICAR was clearly more pronounced during the first 10 hours post injection (Figure 4C and D). RER was also profoundly affected in diabetic rats. In fact, RER of STZ rats was consistently reduced throughout the day and actually eliminated the typical differences that exist between light and dark cycles in control rats (Figure 4C and D). The average RER of STZ rats was 0.860±0.002 and 0.854±0.002 during the light and dark cycles, respectively. RER was further reduced upon treatment of STZ rats with AICAR, reaching average values of 0.822±0.004 during the light cycle and 0.826±0.003 during the dark cycle (Figure 4C and D). These data clearly show that AICAR induced a shift towards whole-body fat oxidation not only in non-diabetic but also in STZ rats. Energy expenditure expressed as heat did not differ during the light and dark cycles in control and AICAR-treated rats (Figure 4E and F). However, in STZ rats, energy expenditure was significantly reduced by 14% and 20% during the light and dark cycles, respectively, when compared to controls. Treatment of STZ rats with AICAR raised energy expenditure of these animals to values similar to those of control rats (Figure 4E and F). Analysis of spontaneous ambulatory activity also revealed no differences during the light and dark cycles between control and AICAR-treated rats (Figure 4G and H). In STZ rats, ambulatory activity was significantly reduced by ∼51% and 48% during the light and dark cycles, respectively, when compared to control rats (Figure 4G and H). AICAR treatment raised ambulatory activity of STZ rats to values similar to those of control rats during the light cycle. AICAR also increased dark cycle ambulatory activity of STZ rats, but it still remained 25% lower than control values (Figure 4G and H).

### Content and Phosphorylation of AMPK, ACC, STAT3, and SOCS3 in the Hypothalamus

**Figure 5:**
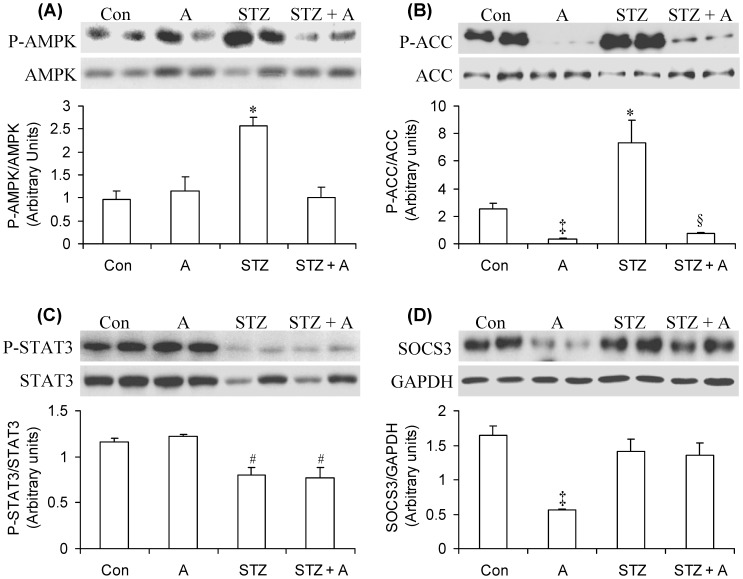
AICAR treatment reduces AMPK and ACC phosphorylation but does not prevent the reduction in STAT3 content and phosphorylation in the hypothalamus of STZ rats. At the end of the study, hypothalami were quickly dissected from control (Con, saline-injected), AICAR-injected (A), streptozotocin (STZ), and STZ plus AICAR (STZ+A) rats and used for western blotting. Representative blots and respective densitometric analyses of tissue homogenates probed for phosphorylated and total AMPK (A), phosphorylated and total ACC (B), phosphorylated and total STAT3 (C), total SOCS3 and GAPDH (D). *P<0.05 vs. Con, A, and STZ+A. ^#^P<0.05 vs. Con and A. ^‡^P<0.05 vs. Con STZ, and STZ+A. ^§^P<0.05 vs. Con, A, and STZ. (One-way ANOVA, N = 4).

Western blotting analysis revealed that control and AICAR rats had similar rates of hypothalamic AMPK phosphorylation (Figure 5A). However, in STZ rats, this variable was significantly increased by ∼1.9-fold and treatment of STZ rats with AICAR reduced hypothalamic AMPK phosphorylation to values similar to those of control rats (Figure 5A). Even though hypothalamic AMPK phosphorylation was unaltered in non-diabetic rats treated with AICAR, ACC phosphorylation was found to be markedly reduced in these animals (Figure 5B). Furthermore, while ACC phosphorylation increased by ∼3-fold in the hypothalami of STZ rats, treatment of these animals with AICAR potently reduced this variable to values equivalent to ∼28% of those of control rats (Figure 5B). STAT3 phosphorylation did not differ between control and AICAR rats, although it was significantly diminished by ∼32–34% in STZ and STZ +A rats. The hypothalamic content of STAT3 was also clearly reduced in STZ and STZ+A rats and AICAR did not affect this variable (Figure 5C). AICAR treatment significantly reduced by ∼67% hypothalamic SOCS3 content in non-diabetic rats, although it did not exert any effect on this variable in STZ rats (Figure 5D).

### Circulating Insulin and Leptin

**Figure 6:**
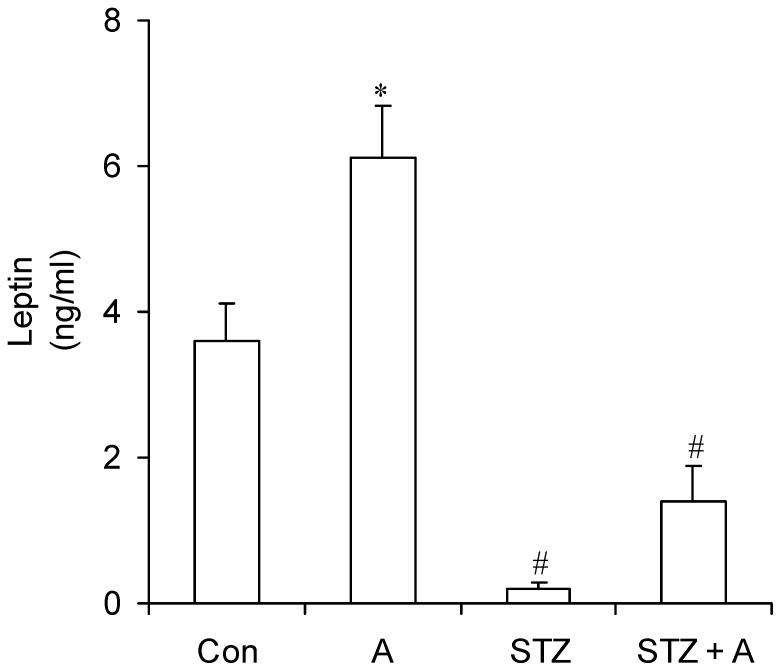
Circulating leptin is significantly increased in AICAR-treated non-diabetic rats, but not in STZ-induced diabetic rats. Blood was collected at the end of the treatment period from control (Con, saline-injected), AICAR-injected (A), streptozotocin (STZ) diabetic, and streptozotocin plus AICAR (STZ+A) rats and subsequently assayed for leptin. *P<0.05 vs. Con, STZ, and STZ+A; ^#^P<0.05 vs. Con and A (One-way ANOVA, N = 8).

As we have previously reported [14], insulin was almost undetectable in the blood of STZ rats, which were also severely hyperglycemic (29.8±0.8 mmol/l). Treatment of STZ rats with AICAR did not cause any improvement in glycemia or insulinemia in these animals [14]. With regards to serum leptin levels, we found that non-diabetic AICAR-treated rats had ∼1.7-fold higher levels of this hormone than control rats (6.11±0.72 ng/ml vs. 3.60±0.51 ng/ml). Conversely, leptin was almost undetectable (0.21±0.06 ng/ml) in the serum of STZ rats (Figure 6). In STZ+A rats, leptin levels were slightly higher than in STZ animals, although not reaching statistical significance (Figure 6).

## Discussion

Here, we provide novel evidence that 7 consecutive days of *in vivo* AICAR treatment attenuated the loss of lean and fat masses while increasing spontaneous physical activity and whole-body energy expenditure in insulin-deficient rats. In non-diabetic rats, AICAR increased circulating leptin, reduced hypothalamic SOCS3 content, and exerted an anorexic effect. In insulin-deficient rats, hypothalamic AMPK and ACC phosphorylation were markedly increased and AICAR treatment completely reversed this effect; however, it was not sufficient to prevented hyperphagia in these animals. This was likely due to the fact that while SOCS3 remained unaltered, STAT3 content and phosphorylation were markedly reduced in the hypothalami of insulin-deficient rats, and these were not altered by AICAR treatment.

The anti-catabolic effects of AICAR on skeletal muscles of STZ rats were fiber type-specific, since only muscles with relatively high oxidative capacities (Sol and EDL) from STZ+A rats had significantly higher mass values than those of STZ rats. Activation of AMPK is supposed to trigger catabolic processes to increase intracellular ATP availability while switching off energy-demanding anabolic processes such as protein sysnthesis [7], [26], [27]. Therefore, it seems counterintuitive that the treatment with an AMPK-activating agent would actually reduce muscle wasting in insulin-deficient rats. However, we have also recently reported that treatment of STZ-induced diabetic rats with AICAR actually attenuated AMPK phosphorylation in EDL and Epit muscles of these animals [14]. It appears that the increase in glycogen content promoted by AICAR treatment [14] minimized the stress of glucose deprivation in EDL and EPI muscles of STZ rats, which in turn attenuated the potential catabolic effects of glucose deprivation-induced AMPK activation. Additionally, since glycogen is highly hydrated and associated with three times its own weight in water [28], the AICAR-induced increase in skeletal muscle glycogen content can previously reported in STZ rats [14] can, at least partially, account for the higher LBM found in AICAR-treated STZ rats. Also, for the maintenance of BW despite the loss of fat found in control rats treated with AICAR. STZ rats were also lethargic and showed significantly reduced ambulatory activity and 24 h energy expenditure; however, these conditions were all reversed with AICAR treatment. Thus, it appears that the anti-catabolic effects of AICAR on skeletal muscle had a favorable impact on spontaneous physical activity and improved the ability of the rats to cope with the dysfunctional alterations in glucose and whole-body energy homeostasis caused by STZ-induced diabetes.

In this study we also show that AICAR exerted fat depot-specific sparing effects in STZ rats, which was characterized by attenuation of mass depletion in the SC Ing but not the Retro fat depot in these animals. We have previously reported that the epidydimal fat pad was drastically reduced in STZ rats and that AICAR treatment also attenuated this effect [14]. These fat-sparing effects of AICAR were only found in STZ rats, since fat mass of non-diabetic rats receiving AICAR was actually reduced, a finding also previously reported by us [18] and others [12]. The fat-reducing effects in non-diabetic rats can be explained by the fact that AICAR caused a 28% reduction in food intake in these animals when compared to controls. AICAR treatment increased circulating leptin and markedly reduced hypothalamic ACC phosphorylation and SOCS3 content without affecting hypothalamic AMPK phosphorylation in non-diabetic rats. These findings are consistent with the suppression of food intake observed in these animals. However, since plasma levels of leptin are directly proportional to fat mass, it is surprising that leptin was increased in the blood of non-diabetic rats that reduced adiposity upon AICAR treatment. In previous studies in which rats received daily AICAR injections for up to 4 and 8 consecutive weeks, we found that adiposity and leptinemia were both consistently diminished in these animals [18]. In spite of that, rats chronically treated with AICAR showed an exacerbated anorexic response to exogenous leptin, which indicated increased sensitivity to this hormone [18]. In this study, rats received AICAR injections for a much shorter period of time (7 consecutive days only) and this could be the reason for these apparently discrepant findings with regards to leptinemia. It is possible that AICAR initially increases circulating leptin and potentiates the anorexic response to this hormone. However, with continued treatment and as significant reductions in adiposity take place, circulating levels of leptin actually drop and reduce the anorexic response to AICAR treatment. In fact, we have previously reported that the anorexic effect of AICAR was much more pronounced in the initial stages (1 to 2 weeks) of treatment with this drug and became progressively attenuated as the treatment extended towards 8 weeks [18]. This could indeed reflect a time-dependent effect of AICAR treatment on adiposity and leptin release. However, additional studies are required to further explore these possibilities.

STZ rats had markedly elevated levels of hypothalamic AMPK and ACC phosphorylation, a condition that was accompanied by a 2-fold increase in food intake when compared to control rats. Also, these animals had drastic reductions in adiposity and almost undetectable levels of leptin in the circulation. Previous studies have demonstrated that leptin inactivates AMPK in the hypothalamus, which, in turn, causes dephosphorylation/activation of ACC and leads to an anorexic response [19], [29]. This is consistent with our findings that hypoleptinemic STZ rats actually had increased hypothalamic AMPK and ACC phosphorylation and were clearly hyperphagic. ACC phosphorylation/de-activation reduces malonyl-CoA synthesis and low levels of this lipid intermediate in the hypothalamus have been associated with increased food intake [30], [31]. Surprisingly, we found that STZ rats remained hyperphagic even though hypothalamic AMPK and ACC phosphorylation were markedly reduced by AICAR treatment. This demonstrated that the activation of ACC in the hypothalamus of insulin- and leptin-deficient rats was not sufficient to inhibit food intake, since alterations in other key signaling steps involved in the regulation of feeding behavior could also be drastically affected in these animals. In fact, further analyses revealed that STAT3 content and phosphorylation were significantly reduced in STZ rats. STAT3 plays a major role in mediating the effects of anorexigenic signals in the arcuate and paraventricular hypothalamic nuclei [19], [29]. Therefore, reduced content and phosphorylation of STAT3 in the hypothalamus must have allowed orexigenic signals to prevail even though AMPK and ACC phosphorylation were potently reduced by AICAR treatment in STZ rats. Furthermore, a major difference between control rats that did elicit an anorexic response to AICAR and STZ rats that did not was that the hypothalamic content of SOCS3 in the former was significantly reduced while in the latter it was not. SOCS3 is a feedback inhibitor of the Jak-Stat pathway that prevents STAT3 activation. SOCS3 overexpression in the hypothalamus induces leptin resistance and obesity [32], while its inactivation in the hypothalamus has been shown to enhance the response to endogenous satiety signals [33] and attenuate obesity in mice fed a high-fat diet [34]–[36]. In this context, it appears that the combination of reduced STAT3 and elevated SOCS3 content prevented AICAR treatment from inducing an anorexigenic response in STZ rats. This indicates that the anorexic effects of systemic AICAR administration are lost under conditions of deficiency of the two major satiety hormones insulin and leptin. However, since AICAR rapidly appears in cortical regions of the brain when administered systemically (i.p. and i.v.) to rats and increases the concentration of adenosine metabolites in these areas [37], we cannot discard the possibility that AICAR modulates the content and activity of STAT3 and SOCS3 independently of leptin and AMPK/ACC signaling in the hypothalamus. In fact, it has been well-documented that hypothalamic functions can be regulated by the activation of adenosine and/or purinergic receptors [38]. In this context, by activating various adenosine and purinergic receptors, AICAR-derived adenosine metabolites could lead to regulation of STAT3, SOCS3, as well as other signaling steps involved in the control of food intake in a leptin- and AMPK/ACC-independent manner. Thus, further studies are required to address these possibilities.

In summary, our data provides novel evidence that the AMPK activator AICAR exerted muscle- and fat-depot-specific anti-catabolic effects and increased spontaneous physical activity and energy expenditure in insulin-deficient rats. AICAR reduced hypothalamic ACC phosphorylation and SOCS3 content and exerted an anorexic effect in non-diabetic rats. However, AICAR did not affect hyperphagia in insulin-deficient rats, even though it significantly reduced AMPK and ACC phosphorylation in the hypothalamus of these animals. These findings indicate despite not preventing hyperphagia, the systemic effects of AICAR treatment ameliorated some of the dysfunctional metabolic alterations of T1D, which could be beneficial for the treatment of this disease.
